# A Case Study of Early Diagnosed Angelman Syndrome: Recognizing Atypical Clinical Presentations

**DOI:** 10.7759/cureus.39271

**Published:** 2023-05-20

**Authors:** Han Dang, Sandhya Srinivasa, Sun Young Lee, Clifford Alprin

**Affiliations:** 1 Pediatrics, University of the Incarnate Word School of Osteopathic Medicine, San Antonio, USA; 2 Family Medicine, North San Antonio Healthcare Associates, San Antonio, USA

**Keywords:** ube3a gene, global developmental delay (gdd), failure to thrive, generalized hypotonia, angelman syndrome

## Abstract

Angelman syndrome (AS) is a rare pediatric neurological condition in which patients most commonly present with inappropriate laughter, microcephaly, speech difficulties, seizures, and movement disorders. AS can be diagnosed clinically and confirmed with genetic testing. In this case report, the patient presented with 9.3% weight loss at two days of age. Although there were multiple attempts at lactational counseling and nutritional guidance, the patient was admitted to the hospital due to failure to thrive. Due to continued global developmental delay and upper and lower extremities hypotonia by the age of nine months, the patient was referred to a neurologist. Brain MRI was negative, and genetic testing revealed 15q11.2q13.1 deletion, which is consistent with AS. Through different therapies and intervention, the patient showed slow improvements in symptoms. This case illustrates the importance of early recognition of nonspecific clinical manifestations of AS. The general management for all AS patients includes physical therapy, speech therapy, mobility support devices, education, and behavioral therapy as they progress through life. Establishing an early diagnosis has potential long-term benefits of improved quality of life and outcomes for patients via early interventions such as physical therapy starting at the age of six months to improve gross motor function. When infants present with nonspecific clinical presentations such as failure to thrive and hypotonia, clinicians should maintain a lower threshold for suspecting genetic conditions, which will facilitate early diagnosis of AS.

## Introduction

Angelman syndrome (AS) is a neurodevelopmental syndrome, which has a prevalence of one in 12,000 to 24,000 [1]. Patients often present with speech abnormalities, developmental delay, movement abnormalities such as ataxia, excessive excitability, and inappropriate paroxysms of laughter. Features present in 80% of AS patients include seizures, which primarily present before the age of three, and microcephaly, which presents before the age of two. Other physical features that may be present in AS include facial dysmorphisms such as prognathia, flat occiput, and tongue protrusion. Clinical presentations that are seen in around 20-80% of AS patients may include, but are not limited to, hypopigmentation, sleep difficulties, scoliosis, and feeding difficulties [2].

AS is most often caused by deletion of* *the *UBE3A* gene on maternally inherited chromosome 15 and silencing of the paternal gene [3]. This affected gene codes for a protein that plays a role in ubiquitination to degrade proteins. There are many genetic variants for AS, with the most common being the deletion of 15q11.2-q13 [2].

The median age of diagnosis of AS is around six and a half years of age when patients start manifesting more recognizable behaviors [4]. A potential explanation for the delayed diagnosis of AS could be due to pediatricians further investigating other conditions with similar manifestations of AS. AS is incurable and managed symptomatically. The multidisciplinary team includes an occupational therapist, a physical therapist, and a speech therapist. Due to the risk of seizures, patients are prescribed anti-epileptics and monitored for seizure activity. As a result, AS patients require lifelong therapy and family support.

## Case presentation

**Figure 1:**
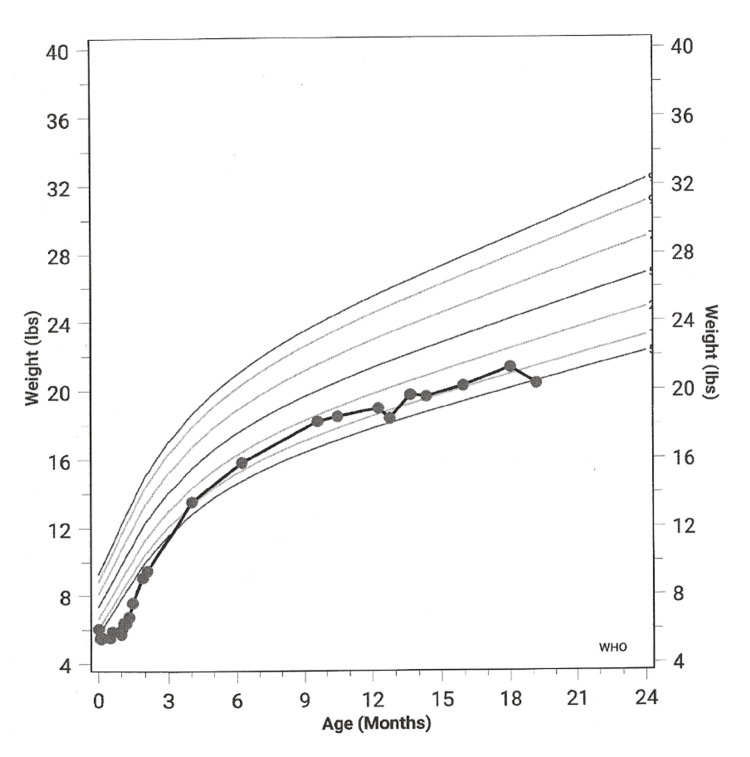
Patient's weight (lbs) for age (months) from birth to 19 months

The patient, a male newborn, was born at 34 weeks’ gestational age via normal spontaneous vaginal delivery without any complications. As shown in Figure 1, the patient’s birth weight was 6.1 lbs, and by two days of age, 9.3% weight loss was noted. Newborn head circumference was normal, measuring 33.4 cm (80th percentile), with no evidence of microcephaly. Since hospital discharge, the patient was nursing on demand with around 30 mL on average of expressed breast milk per feeding. The newborn’s mother denied any problem with her breast milk production and she hears active swallowing during feeding. However, even with lactational counseling and nutritional guidance, the patient was admitted at one month for failure to thrive due to inability to gain back his birth weight. After hospital admission, the patient was referred to pediatric gastroenterology, but yielded negative findings for anatomical and physiologic abnormalities such as obstruction, malabsorption, and malrotation. Parents were instructed to use formula only with proper feeding techniques, and since then the patient was able to gain appropriate weight.

When the patient followed up for a well-child visit at two, four, and six months of age, he did not meet age-appropriate developmental milestones. At the age of two months, the patient did not have a social smile. At the four-month well-child visit, he was only able to partially roll. He was then referred to physical therapy and speech therapy due to suspicion of a developmental pathology. By nine months of age, the patient was diagnosed with global developmental delay and hypotonia.

Due to underlying neurological abnormalities, the 13-month-old patient was referred to a neurologist. Physical examination showed a non-distressed infant with normal head shape and circumference for his age. Atraumatic, coarse facial features were appreciated. There was no evidence of tongue protrusion, flat occiput, prognathia, microcephaly, and tremor-like movements. The patient exhibits no excessive laughter. Hypotonia of upper and lower extremities were noted with 5/5 strength, full range of motion, and 2+ deep tendon reflexes bilaterally. Brain MRI revealed no acute infarct, brain atrophy, intracranial hemorrhage, mass effect, midline shift, extra-axial fluid collection, or hydrocephalus. The patient did not have a history of seizure.

At the age of 13 months, due to unexplained hypotonia, genetic testing was ordered, which revealed the 15q11.2q13.1 deletion consistent with the diagnosis of AS. The patient made slow progress with therapies and the family was made aware of the diagnosis, prognosis, and future complications that can occur with AS.

## Discussion

Early signs of AS such as developmental delay, microcephaly, hypotonia, and failure to thrive are nonspecific. Thus, patients with AS can be misdiagnosed with similar pathologies. These other conditions include cerebral palsy, hypoxic-ischemic encephalopathy, myotonic dystrophy, and cerebral malformations, which yield positive brain MRI findings. Inborn errors of metabolism can present with similar clinical features as well, but metabolic and mitochondrial panels would be positive. Other genetic differential diagnoses include, but are not limited to, *MECP2* duplication syndrome, WAC-related intellectual disability, adenylosuccinate lyase (ADSL), and *TRPM3*-associated syndromes.

*MECP2* duplication syndrome affects males mainly due to its X-linked inheritance pattern. Most males with this syndrome have moderate-to-severe intellectual disability, infantile hypotonia, feeding issues, seizures, and nonspecific anomalies on brain imaging. However, patients with *MECP2* duplication syndrome have recurrent respiratory tract infections, especially pneumonia [5].

In patients with *WAC*-related intellectual disability, more than 75% of newborns or infants present with hypotonia including oral hypotonia, which can lead to feeding difficulties and failure to thrive [6]. However, only 10%-30% of patients with *WAC*-related intellectual disability present with seizures. Seizure is a more common feature seen in AS than in *WAC*-related intellectual disability [7].

ADSL deficiency is characterized by microcephaly, axial and generalized hypotonia, acute encephalopathy with early signs of poor feeding, lethargy, and seizures. ADSL can be distinguished from AS through brain MRI, which may reveal cerebral cortex, cerebellar vermis, and corpus callosum atrophy [8].

In patients with *TRPM3*-associated syndrome, Val837Met recurrent substitution in the *TRPM3* gene, which encodes transient receptor potential cation channels of the melastatin family, is associated with global developmental delay, hypotonia, epilepsy, and severe intellectual disability. Common *TRPM3*-associated syndrome facial features such as deep-set eyes, wide forehead, and downturned mouth are different from the characteristic AS findings of a flat occiput, wide mouth, widely spaced teeth, and protruding tongue [9].

The diagnosis of AS is based on clinical judgment and genetic testing. Our patient has a relatively uncommon presentation of AS. For instance, microcephaly, a common feature affecting 80% of AS patients, was not evident. Moreover, onset of seizures, which presents in 75% of AS patients before the age of three, was also absent in this patient [10]. This case study suggests that pediatricians should have a low threshold of suspicion for diagnosis of AS when infants present with nonspecific symptoms such as failure to thrive, hypotonia, and global developmental delay.

This patient’s MRI was also normal and did not show the characteristic demyelination and atrophy [10]. Other early-onset classic symptoms such as social smile, excessive laughter, or tremor-like movements were not noted in this patient [2]. Thus, it is clear that there is a greater variation in the presentation of patients with AS. Pediatricians should be made aware of the variability, and the presence of severe hypotonia and feeding difficulties should prompt some suspicion for AS. Part of the reason for the earlier diagnosis in this case at 13 months of age was due to the pediatrician and neurologist maintaining some degree of suspicion for AS instead of waiting for the classic symptoms to appear. In order to detect the more obvious signs of AS, pediatricians would have to wait until ambulation is achieved, which is typically at around two years of age. Likewise, any variations in the common presentation of the patient would also contribute to a much later diagnosis. This may account for the median age of diagnosis at six and a half years.

Early diagnosis of AS is beneficial for the patient’s well-being and quality of life. AS patients require not only physical but also emotional support during childhood and transition to adulthood. The patients may require physical, speech therapy, mobility support devices, and educational and behavioral therapies.

Hypotonicity in all extremities leads to decreased mobility, loss of balance, and difficulty in ambulation in AS patients. In the case report by Kara et al., early diagnosis and an early physical therapy program starting at 6 months of age showed promising improvements in the patient's gross motor function. The Berg Balance Scale (BBS) assesses balance and motor ability to help gauge the effectiveness of physical therapy. In Kara et al.’s case report, balance was assessed with BBS in the patient with AS. Before the therapy, the patient had a high risk of falling secondary to hypotonicity; however, with physical therapy, the patient showed improvements over the course of six months [11]. As a result, early diagnosis of AS can be beneficial for patients, specifically to improve hypotonicity.

According to the review by Pearson et al., there is a disassociation of speech and non-verbal communicative behavior in AS patients. This emphasizes the need for early interventions, which can optimize a patient's communication skills by converting nonverbal behaviors into discrete words [12]. The Enhanced Natural Gestures (ENG) developed by Calculator include a variety of natural gestures and sign language for patients with AS. The study included parents of 18 patients with AS who integrated the ENG program into daily interactions with their children. The patients who met or exceeded the program’s expectations in communication and parents found it to be not too difficult to self-administer the ENG program [13]. Therefore, early awareness of the diagnosis and the utilization of ENG program is beneficial for both patients with AS and their families.

AS patients may have normal life expectancy; however, they require a lifelong guardianship. Dependency and constant care can cause caregiver burdens. When family members are made aware of the diagnosis and challenges early, they can benefit from early education about home exercises and in-house arrangements to give the best care and support to AS patients.

## Conclusions

Diagnosing AS early is challenging due to nonspecific symptoms and increased likelihood of misdiagnosis with other common genetic conditions. Delayed diagnosis can potentially lead to worsening of the hypotonia and speech difficulties. Combating hypotonia and speech abnormalities earlier can improve balance and ambulation issues as well as allow the patients to develop their own communication styles. This report is a good example for giving clinicians a lower threshold for the suspicion and diagnosis of AS. This can potentially lead to an earlier age of diagnosis and better outcomes for AS patients.
